# The light, the dark, and everything else: making sense of young people's digital gaming

**DOI:** 10.3389/fpsyg.2023.1164992

**Published:** 2023-06-12

**Authors:** Mikko Meriläinen, Maria Ruotsalainen

**Affiliations:** ^1^Game Research Lab, Tampere University, Tampere, Finland; ^2^Department of Music, Art and Culture Studies, University of Jyväskylä, Jyväskylä, Finland

**Keywords:** digital gaming relationship, young people, digital gaming, social worlds, everyday life, transmedia, thematic analysis

## Abstract

Whether gaming has a beneficial or detrimental effect on young people's lives is a defining feature in both the research and the public discussion of youth digital gaming. In this qualitative study, we draw from a thematic analysis of the experiences of 180 game players in Finland, aged 15–25 years. Utilizing the digital gaming relationship (DGR) theory, we explore how different aspects of gaming actualize in their lives, and how different features of gaming culture participation come together to form their experience. We contend that framing gaming as a balancing act between beneficial and detrimental obscures much of the complexity of young people's gaming, reinforces a partially false dichotomy, and overlooks young people's agency. Based on our results, we suggest alternative approaches that help reduce and avoid these problems.

## 1. Introduction

Young people actively participate in gaming cultures: in Finland, where this study took place, 76.2% of 10–19-year-olds play digital games weekly and 42.2% do so daily, while in the 20–29 years age bracket, the corresponding percentages are 66.7 and 24.4% (Kinnunen et al., 2022), respectively. As they live in a world where gaming is a part of everyday life, it is not surprising that for many young people, gaming is an important part of identity development (Granic et al., 2020) and social life (Bengtsson et al., 2021).

The impact of games and gaming cultures on young people's wellbeing and behavior has been a source of concern and interest from the early steps of modern digital gaming in the late 1970s and early 1980s to contemporary times (Rogers, 2013). However, research on young people's gaming has predominantly focused on their game play, typically through exploration of variables such as time spent playing and game play motives, and their connections to various outcomes regarding, for example, psychological wellbeing or learning, usually applying quantitative methods (e.g., Hamre et al., 2022). A minority of individual, usually qualitative, studies have focused on specific aspects of youth gaming, such as gaming as part of social life and friendships (e.g., De Grove, 2014; Eklund and Roman, 2019; Bengtsson et al., 2021), or youth views on problematic gaming (Nielsen, 2016), gaming-related parenting (Meriläinen, 2021), online gaming conduct (Kaye et al., 2022), or game content (Kutner et al., 2008). To our knowledge, only a few studies (Lenhart et al., 2008; Aarsand, 2012) have sought a more general understanding of young people's relationship with gaming.

Gaming is often seen as a dichotomous activity defined by the sometimes stark contrast between its “dark” and “light” sides (e.g., Greitemeyer, 2022), and issues such as violent content and problematic gaming are juxtaposed with aspects such as friendship, learning, and relaxation. However, many aspects of gaming sit outside the dichotomy of clearly beneficial or detrimental, light or dark, but are at the core of gaming nevertheless.

In this article, utilizing the digital gaming relationship (DGR) theory (Meriläinen, 2023; see also Sokka, 2021) as a lens, we draw on young people's views on gaming to broaden perspectives on youth gaming. Based on our analysis, we suggest alternative research perspectives to construct a more complete picture of young people's gaming. We wish to point out that our use of the word “gaming” in this article does not refer only to the act of playing games, covering instead a diverse constellation of gaming culture activities (Kahila et al., 2021). Gaming is participation in the social world of digital gaming, a socially constructed sphere of interest and involvement that individuals engage with varying intensity and attachment, and is influenced by social, personal, and cultural and societal factors (Unruh, 1979; Meriläinen, 2023). Contemporary games are often transmedia products (Koskimaa et al., 2021), and their fiction unfolds through multiple mediums, inviting varied forms of engagement.

## 2. Background

Much of previous research on youth gaming has focused on the impact of gaming, typically examined through variables such as gaming motives and spent time, on different aspects of wellbeing. While causality often remains unclear, gaming has been connected to a wide range of beneficial and adverse phenomena, from enhanced working memory and task-related brain activity (Moisala et al., 2017) and academic learning (Martinez et al., 2022) to depressive and musculoskeletal symptoms (Hellström et al., 2015).

Research focusing on youth gaming seen as problematic or disordered (e.g., Van Rooij et al., 2011; Männikkö et al., 2017; Su et al., 2018; Chang et al., 2022) has been prominent and increasing since the early 2010s, sparking considerable debate (see Aarseth et al., 2017 and responses) and making up a considerable part of contemporary research on young people's gaming. As a phenomenon, problematic gaming exemplifies the often blurred lines between genuine risk and moral panic that prominently feature in research on youth technology use and associated public discussion and policy decisions (see Rogers, 2013; Orben, 2020). The discussion of problematic gaming, coupled with the long-running academic and public debate regarding the potential impact of violent game content on aggressive behavior (see Mathur and VanderWeele, 2019) and contemporary worries over so-called screen time (Orben, 2020), has firmly grounded research and public discussion on youth gaming in risk perspectives, with the benefits of gaming offered as a counterbalance (e.g., Granic et al., 2014; see Behrenshausen, 2012).

While the risks of gaming have been widely documented, many studies (e.g., Männikkö et al., 2017; Hamre et al., 2022) on the impacts of gaming note that outcomes are contingent on a wide range of variables: they typically do not stem from gaming as such, but rather from an interplay of ways of engagement, motives, life situations, time spent, and games played, as well as factors such as gender, race, and age. As a result, discussing young players as a more or less homogenous group erases these fundamental differences. Our rationale for this study springs from this diversity; to better understand young people's gaming, we have to explore individual experiences and narratives to avoid collapsing vastly different experiences into generalized categories and stereotypes. To do this, we apply the DGR theory, discussed next.

The DGR theory examines individuals' engagement and relationship with the social world of digital gaming and was developed from the sport sociological theory of physical activity relationship (Koski, 2008) by Sokka (2021) and expanded by Meriläinen (2023). As a new theory, it has shown promise as a tool to understand gaming as a complex phenomenon (Meriläinen, 2023). The theory takes as its starting point that each individual has a different relationship with gaming, and an individual's relationship with gaming develops, and is actively constructed, over a long period of time and is influenced by a variety of factors. These factors are the personal meanings given to gaming, internal and external influences on gaming, different ways of engaging with gaming, and the level of engagement with gaming. The DGR theory acknowledges that there are as many individual formulations of young people's gaming as there are young people, and thus lends itself well to the qualitative exploration of individual experiences while also allowing the identification of wider phenomena.

## 3. Data and method

The data used in this study are a set of responses to a Finnish language qualitative online questionnaire (see Supplementary File 1) constructed by the first author and consisting of seven voluntary, open-ended questions and background questions (age, gender, cultural background, and living region), collected in Finland between May and June 2021. The questionnaire was targeted at 15–25-year-old Finnish speakers who played digital games. The questionnaire link was shared on social media (*Twitter, Facebook*, and *Discord*) through both professional and personal networks, with an emphasis on specific groups such as Discord gaming communities and Facebook groups for professionals, such as teachers, youth workers, and media educators, working with young people, requesting that they share the questionnaire to young people they work with. Several actors, both individuals and organizations, in the fields of gaming, academia, youth work, and media education also distributed the questionnaire through their public accounts. This resulted in a self-selected sample of 180 respondents.

The seven main questions were intentionally broad (e.g., “Does something limit your gaming?”) to avoid constraining the responses. However, to assist participants, we provided several example subquestions (e.g., “Do your parents set limits on your gaming?” and “Do you avoid certain games or communities?”). It was explicitly stated in the questionnaire that these subquestions were examples to help respond to the broader questions and that respondents were not required or expected to address the subquestions. Most respondents (*N* = 163, 90.6%) answered all seven main questions. As all questions were voluntary, we also included responses (*N* = 17, 9.4%) in which the respondent had answered some questions. Individual answers to the questions varied considerably in length: some consisted of a single word, while others ran for several paragraphs.

The whole age range of the target group (15–25) was represented in the data, with an average age of 20.6 and a median of 21 years. Out of the 180 respondents, 120 (66.7%) were men, 46 (25.6%) were women, and 11 (6.1%) were non-binary. Three respondents (1.6%) elected not to disclose gender information. While all ages in the 15–25 years range were present in the men's sample with an average age of 20.1 years, the youngest woman to respond was 17 years and the average age in the women's sample was 21.8 years. The small non-binary sample fell between these two, with an average age of 20.8 years.

Nearly all respondents (*N* = 177, 98.3%) were born in Finland, with 11.1% of the respondents (*N* = 20), reporting that one or both of their parents were born in another country. Very few respondents reported belonging to a cultural or language minority; five respondents (2.8%) were Swedish-speaking Finns and two respondents (1.1%) were Sámi.

We conducted a thematic analysis (see Braun and Clarke, 2021) on the responses, utilizing a combination of data and theory-driven approaches. We familiarized ourselves with the responses and, using the qualitative analysis software *Atlas.ti*, coded the responses, first individually and then together, going over the responses several times, coding and re-coding, combining notes, and discussing until we reached an agreement.

In the coding process, we identified different aspects that we considered relevant and interesting, to capture a detailed and diverse picture of young people's gaming. After removing redundancies and merging overlapping codes, the process resulted in 437 individual codes that ran from single mentions (e.g., “Worry over too much sitting”) to broad topics mentioned by the majority of the respondents (e.g., “Gaming with friends”). Next, we grouped codes to make larger subthemes (e.g., “Gaming and gender” and “Public discussion of gaming”), experimenting with a variety of configurations. We then looked at these groupings through the lens of the DGR theory, as discussed next.

## 4. Results

**Figure 1:**
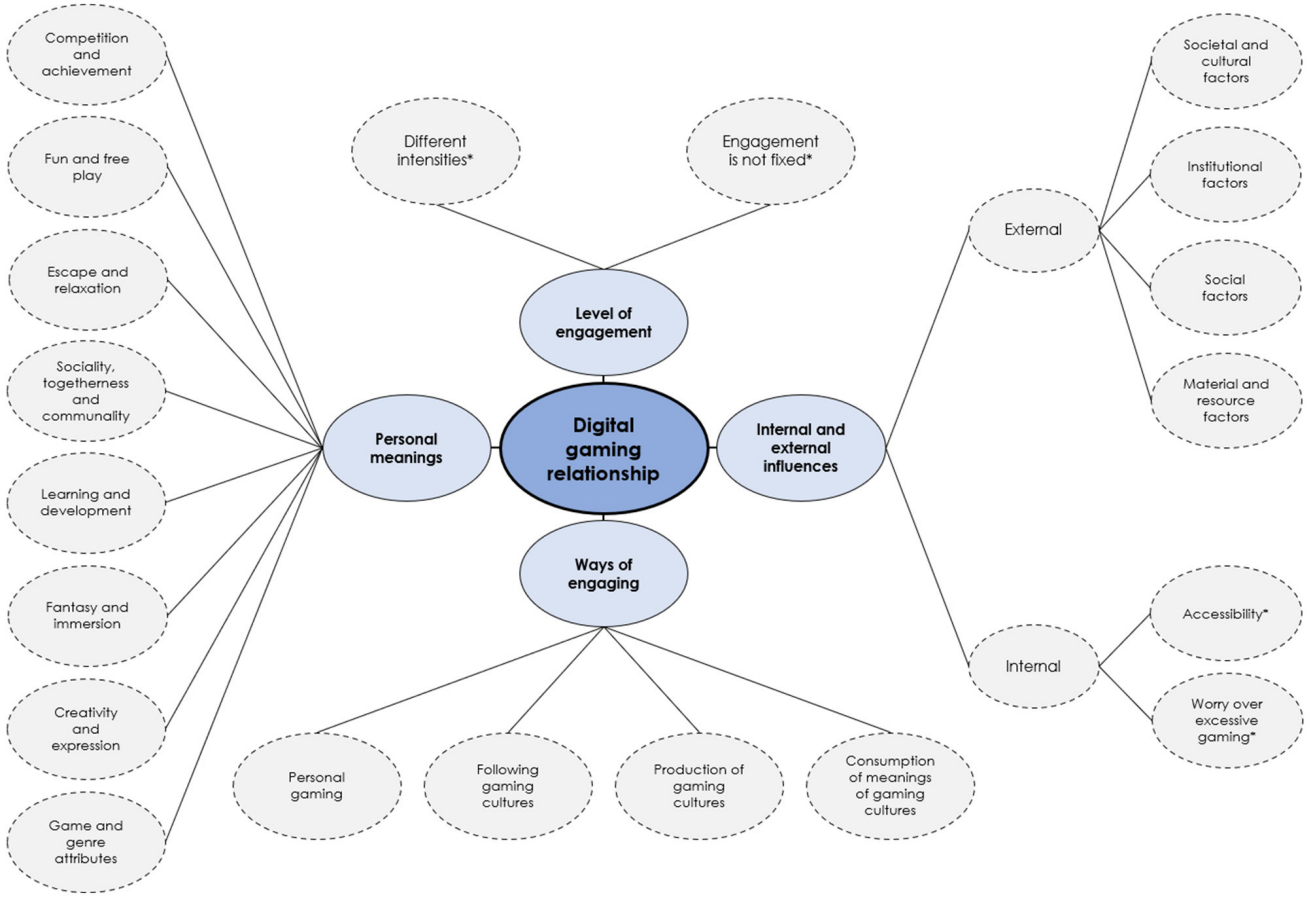
Thematic map of main themes and subthemes. Asterisk denotes a subtheme not derived from the DGR theory.

We present our analysis organized by the four main themes derived from the DGR theory (Figure 1). We start with the *Level of engagement* to demonstrate the considerable differences in young people's engagement with game cultures, followed by the *Personal meanings of gaming*. We then explore the *Internal and external influences* that shape individuals' relationship with gaming and conclude with the different ways gaming takes place in the theme *Ways of engaging with gaming*.

We have illustrated our themes with quotes from the responses, translated from Finnish. For ease of reading, we have made minor grammar and punctuation changes to some of the quotes during the translation. Because of the contested nature of the word “gamer” (see Shaw, 2012; Paaßen et al., 2017), we have used the word “player” when referring to someone who plays games.

### 4.1. Level of engagement

This theme consisted of two subthemes: *Different intensities* and *Engagement is not fixed*. The first focused on the different levels of engagement based on Unruh's (1979) formulation of social world participation, whereas the second addressed the shifting nature of this participation.

#### 4.1.1. Different intensities

In terms of the DGR theory and Unruh's four types of social world participants (*stranger, tourist, regular*, and *insider*), all types of participation could be identified. The vast majority of respondents could be classified as *regulars*: they routinely participated in gaming, displayed attachment to it, and had regular company to play games with.

CS:GO [*Counter-Strike: Global Offensive*] has been extremely important for me ever since I ran into the game. It's been a tool to stay in contact with friends who live in other cities. Gaming is an important way for me to support social relations, that would be otherwise be almost impossible to sustain. Because the gaming itself is not the whole thing, but simple things like going through what's happened during the day is also a part of it. … Watching gaming streams and YouTube has replaced TV and other streaming services for me. Man, 20

In contrast to the teenagers interviewed a decade earlier by Aarsand (2012), these young people did not appear to strategically position themselves as “ordinary players” by distancing themselves from “casual” or “hardcore” players—possibly owing to the different research methods (anonymous online questionnaire vs. face-to-face interviews) and the further normalization of gaming since Aarsand's research (see also Vahlo and Karhulahti, 2022). Gaming appeared as a mundane part of their everyday life despite sometimes very intense gaming. However, when asked about negative gaming experiences or worries over gaming, some respondents specifically mentioned that gaming was not a problem for them if they were discussing intensive gaming, aware that it could be interpreted as such.

Gaming has never been a problem for me. I've played for long periods in a row, but I can be without the computer and games too. Like driving around with friends. But I spend my free time home and at the computer, and quite often gaming. This doesn't have a big negative impact on my life. Woman, 22

A minority of respondents could be seen as *insiders*, expressing the importance of and their belonging to the social world of gaming in different ways. Below are two very different examples.

In terms of players, I'm a so-called “hc” (hardcore) player which means that I game a lot using a purpose built computer. … Attitudes towards games are nowadays pretty open because almost everyone plays to some degree. In a way [this is also] a bad thing because many casual players don't have a clue about gaming culture in general and they don't care about the actions of game developers so they're easy to exploit to hammer out a profit. They're also often shocked about harsh language and demand game developers to address it, which just makes the whole culture/hobby even worse. Man, 22

I encourage so-called noobs [new players] and offer advice whenever possible. I automatically block rude players and report inappropriate behaviour. … I run my own clan in *Warframe* and organize events and LAN parties. Woman, 25

The first comment echoes common discourses in gaming cultures. Although common in marketing and everyday language, the “gamer” or “hardcore gamer” identity is often seen as a negative one defined by exclusivity and hostility and is sometimes actively avoided (Shaw, 2012). The insider status partially differs from Unruh's (1979, p. 120–121) definition: although gaming appears to be an important part of the respondent's identity, the influx of new individuals into gaming appears more as a degradation of gaming culture, instead of a necessity to the social world's existence. Owing to the scale and dispersed nature of gaming, such insiders have few ways of policing access to the social world, which may further feed frustration. In the second quote, the respondent mentions several features pointed out by Unruh, namely the active creation and controlling of the social world through organizing events and reporting inappropriately behaving players and the recruitment of new members by both encouraging starting players and providing ways of entry through events.

At the other end of the engagement spectrum, a single respondent mentioned that they did not play games at all, their gaming culture participation limited to occasionally watching Let's Play videos (videos of commented game play) on *YouTube* (see Orme, 2021). This respondent can be seen as a *stranger* in the social world of gaming. Although engaging with gaming culture, their engagement is ephemeral and uninvested.

The fourth group of respondents were those classified as *tourists*, who enjoyed different aspects of gaming but were nevertheless not very invested in it, transiency and entertainment being common features of their gaming (Unruh, 1979, p. 119). They might regularly play different kinds of games on a variety of platforms and devices or have a history of very intense gaming yet did not view gaming as all that important. This observation shows the shifting nature of DGR and contests common notions of “casual gaming”, stereotypically seen as occasional smartphone gaming limited to a few games (see Juul, 2010).

I play FPS [first-person shooter] games with my friends, but also MMORPGs, as well as driving games and all sorts of roguelike [a subgenre of digital role-playing games] games. I also play single-player games or online games by myself. I play on the computer as well as on gaming consoles and sometimes on the phone. Gaming used to be a part of my everyday life, but nowadays I only play if there's a new trendy game or if I suddenly feel like it. … I also used to watch some gaming youtubers/streamers as my idols, so that's also influenced how I'm growing up/have grown up … If I need to choose between a hobby and gaming, I usually choose the hobby without the slightest thought. Man, 16

#### 4.1.2. Engagement is not fixed

Participation in gaming can change for a variety of reasons. As Bergstrom's (2019; see also Wiik, 2023) exploration of the players of the massively multiplayer game *EVE Online* demonstrates, quitting or actively gaming is not a binary, but a spectrum. In our data, changing life priorities and responsibilities were a common source of change in participation. As demonstrated by the second quote below, a change in participation sometimes impacted only a part of the social world.

Gaming is important to me, and I even consider it a hobby. My gaming fluctuates: there are periods when I don't play at all, at other times periods when I play even several hours per day. Woman, 19

I don't play anything anymore because I see games as too addictive. They fill your mind even when you're not on the computer … I was definitely gaming too much, easily 5–8 h per day. That's far too much. … I watch a lot of streams, esports even though I don't play myself anymore. I like watching *CS:GO*. Man, 22

Changes in gaming did not always imply profound changes in an individual's relationship with gaming. Gaming activities might be stopped for long periods at a time, yet an individual could still view gaming as an important part of their life and participate in the social world. This said, few participants discussed long-term changes in their gaming participation.

### 4.2. Personal meanings of gaming

In this main theme, we examined the eight dimensions of personal meaning as defined by Meriläinen (2023): *Competition and achievement*; *Fun and free play*; *Escape and relaxation*; *Sociality, togetherness, and communality*; *Learning and development*; *Fantasy and immersion*; *Creativity and expression*; and *Game and genre attributes*. Although these dimensions overlap with gaming motives, they are broader wholes that represent respondents' self-positioning in relation to different gaming phenomena (Meriläinen, 2023). As different dimensions of a single broader whole, they intersect and overlap, and are not exclusive categories.

#### 4.2.1. Competition and achievement

Competition is a key feature of many popular games and gaming culture phenomena from early arcades to contemporary esports. It is also a divisive topic that some respondents felt very strongly about. Discussion of this dimension was typically focused on game play mentalities and practices, although it also touched more broadly on competitive gaming culture. Many respondents mentioned playing popular competitive games such as *League of Legends, Overwatch*, or *CS:GO*, and competition and developing gaming skills were brought up by many respondents as an important part of their gaming. Competition was not only about victory over others but also about feelings of success and competence (see Ryan et al., 2006; Vahlo, 2018).

The feeling of winning and success is important, the feeling that you can do this and you're good. Especially when playing against other people the feeling of being better than another person is amplified, not so much when playing against the computer and you're just happy about your own performance. Man, 19

Competition was a dimension where different goals and mentalities sometimes clashed. Respondents reported their annoyance over their fellow players being either too serious or too easy-going, depending on their preferences.

I often play multiplayer games trying to do my best and I'm pretty competitive, sometimes it can be annoying if a friend/teammate is not trying to win and is playing “just for fun”. Man, 21

Respondents discussed not only their preferences but also voiced their views on how they perceived competition as influencing gaming more broadly. Some respondents saw esports and the professionalization of gaming as a reason for gaming becoming too competitive (see Blamey, 2022). Echoing the different gaming mentalities mentioned above, these respondents saw a focus on competition as antithetical to relaxed fun and enjoyment (see Brock, 2017).

Contemporary gaming culture has become remarkably competitive, even excessively so. I for example no longer play FPS game almost at all because good-natured fun has almost completely disappeared from them, and the game is approached like a high-level sport. Non-binary, 18

#### 4.2.2. Fun and free play

Closely related to the previous dimension, *Fun and free play*^1^ addresses gaming for fun and playful experiences. Experiences of fun can be difficult to pinpoint exactly (Vahlo, 2018), and respondents described a wide variety of things that contributed to the fun, from immersion in game narratives to fooling around with friends while gaming.

Although gaming is probably for the most part “fun”, however individuals interpret the word, it is not always about playful leisure, but can, for example, be work (Bihari and Pattanaik, 2023), monotonous “grinding” of repetitive content or systematically working to hone gaming skills (Pargman and Jakobsson, 2008; Vahlo, 2018), or professionally creating video content (Törhönen, 2021). Gaming can also have instrumental functions. Here, the respondent makes a distinction between the instrumental use of gaming as coping, and playing “just for fun”:

I mostly play games for fun, as my hobby, but sometimes also to escape the tumult and stress of life. Man, 22

Having fun and approaching gaming from a playful point of view (see Masek and Stenros, 2021) was seen as normative by some respondents: gaming should be first and foremost fun. One respondent connected hostile behavior to taking gaming too seriously.

I'm most worried about how angry players seem to be at each other nowadays. Or how some people take it [gaming] far too seriously. Man, 23

In some cases, playing for fun was related to the overall lower importance of gaming, echoing the idea of Unruh's (1979) tourist: visiting the world of gaming occasionally for entertainment, but not being very attached to it. As demonstrated by many of the respondents, gaming can hold great personal significance. However, for the majority of players, gaming is likely not a life- or identity-defining or otherwise profoundly important activity, but more about entertainment and killing time, even if they actively play and enjoy games.

I play games because it's something fun to do—usually because of the story and characters. … I think gaming is fun but on no level does it rank over other casual hobbies. I do it when I feel like it, meaning irregularly. Woman, 23

I play to kill time and mostly at home. I usually play because there's nothing better to do. I'm primarily a console player, and play regularly on the PS3, PS4, Switch and 3DS. Although I play a lot, it's not all that important to me, because I'm mainly looking to kill time because of my current life situation. Man, 18

The dimension also encompasses different types of playful behavior both during game play and in gaming culture more broadly, such as cosplay. Trolling in its different forms (Stenros, 2015; Cook et al., 2018) was an interesting example, as some participants did not frame their self-labeled trolling behavior as hostile, but as playful, prioritizing engagement over consequence (see Masek and Stenros, 2021). More hostile forms of trolling are discussed under the subtheme *Sociality, togetherness, and communality*.

I mainly troll in ways that don't really cause harm to anyone. In *Valheim* I like to build treasure chests on the map in the middle of the forest and fill them with either useful or useless items for others to find. Together with another player we built a hut in secret, and filled it with signs with aphorisms written on them. Non-binary, 25

#### 4.2.3. Escape and relaxation

Relaxation, stress relief, and psychological coping are common motives and outcomes for gaming (e.g., Ryan et al., 2006; Snodgrass et al., 2014; Caro and Popovac, 2020), and for many respondents, gaming was a way of supporting psychological wellbeing and served as a counterbalance to stress, for example through gaming as a comfortable routine, intense pleasant emotional experiences, or spending time with friends. The apparent instrumentality varied: some respondents mentioned that gaming was relaxing, whereas others explicitly reported using gaming to relax and relieve stress.

Several respondents formulated a distinction between gaming and other aspects of everyday life, and described this as an “escape.” Despite this figure of speech, gaming appears very much positioned in the midst of everyday life with its associated problems and responsibilities: the figurative escape is, for example, comfort and time to oneself.

For me, games are an opportunity to escape the rest of my life for a moment, and amidst an ending romantic relationship or health problems gaming has been a source of comfort and joy. Woman, 21

The opportunities for relaxation that gaming provided were also seen as a risk. Echoing previous research (Snodgrass et al., 2014; Shi et al., 2019), the easy escape that gaming provided was seen as problematic. Illuminating the problems of dichotomous approaches, both beneficial and detrimental qualities could appear simultaneously, and players were aware of this.

I'd say that in terms of my quality of life it would be better for me to more often do something else than play games. Games often offer only short-term pleasure and filler for my life and they don't really result in long-term benefits like reading or just leaving the house in general might. Of course I think gaming with my friends is important, but I'm specifically talking about gaming I do by myself. Often gaming is an “easy” solution and deep in my comfort zone and I should try to leave it considerably more often. Man, 25

#### 4.2.4. Sociality, togetherness, and communality

Gaming is a common part of many young people's social life (e.g., De Grove, 2014; Eklund and Roman, 2019; Bengtsson et al., 2021), and different aspects of social participation in gaming featured prominently in the data. Social interactions, or lack thereof, could considerably influence an individual's gaming experiences (see Kaye and Bryce, 2012) and relationship with gaming, for example through a sense of community and belonging, avoiding interaction with other players, experiences of discrimination, or conflict with friends and family members. None of the responses suggested that the respondent's gaming was completely asocial. Even those who preferred single-player games, or for whom gaming was very private, still participated in online communities related to their favorite games, visited events, or discussed games with their friends.

Video games have been the source of my sociability since my youth. My friends at school were those who I gamed with and I could talk about things while gaming. I got to know my first girlfriend because she was wearing the shirt of a Youtuber I liked (Laeppavika [Finnish gaming Youtuber]). Man, 22

I play games alone because I can't be bothered to deal with other people when I just want to relax with a game. Looking at [game] culture as a bystander, it feels like I've made the right choice and I wouldn't ever bother to try gaming especially with strangers. … Gaming is a part of interacting with many of my friends and at the very least they've been a part of building friendships or [friendships] have even been born from them. Non-binary, 23

Online conduct was often commented on, and online hostility (see Kowert, 2020) was a major problem discussed, and sometimes participated in, by the respondents. An important observation is that there was not a clear definition between a “toxic” and “non-toxic” player: respondents describing their online conduct as friendly and helpful could also lose their cool in the heat of a gaming situation. Respondents paid attention to their own conduct as well and sought to regulate it out of consideration for others.

In general what annoys me in gaming culture is the amount of hate speech and harassment. I'm for example not at all surprised why women may find gaming communities repulsive. I try to make my own gaming environment a safe space for everyone. Man, 25

Can I be polite? Yes, if I'm playing games like for example *VRChat*, in which there's no competitive value, so it's much nicer to get along than make yourself hated, especially in a game where there's nothing else than talking with others. *CS:GO* always crosses the line, in intense competitive games like that it's difficult to have fun otherwise. I've been in tears with laughter several times just because another player gets upset by my and many of my friends' shit talking. Shit talk is part of gaming, you can't eliminate it. The same applies to for example basketball or especially boxing/UFC. Man, 16

I do my best to cheer on other players and keep up a positive gaming attitude. If I'm too agitated because of something (for example an opposing team's tricks or if the game is lagging) I keep quiet, because I don't want to say anything mean to anyone. I don't however feel like I lose my cool easily, instead there are usually some other factors behind it that lead to my annoyance. Non-binary, 19

#### 4.2.5. Learning and development

As discussed in the background section of this article, learning is often mentioned as an important gaming benefit, and the learning dimension was brought up by many of the respondents. Many respondents, who typically spoke Finnish as a first language, assigned great importance to gaming as a way of learning English as a foreign language especially when they were younger. Respondents also mentioned more abstract development, such as new insight into their behavior.

Games have also in some cases helped with school. E.g., I learned English very quickly because I constantly needed it with games. Woman, 22

I've learned all sorts of things from games and gaming, but they've developed my emotional skills especially. Through games I've found sides to myself that outside gaming I haven't realized or revealed to others. For example when I was younger, aggressive behaviour after the emotional rush caused by gaming was something I never would have believed of myself in the outside world. Nowadays I can deal with the emotions caused by the game in a more “civilized” manner. Man, 25

While gaming was perceived as helping learn a variety of skills, many respondents also discussed the skill of playing games. When considering games and learning, this is a crucial point: rather than a stepping stone for learning something else, gaming can in itself be a skill with personal, social, and professional value, comparable to skills such as playing an instrument or a traditional sport (e.g., Huang et al., 2017; Karhulahti, 2020). Gaming skills and knowledge of different aspects of gaming culture could also be a source of game culture capital (Consalvo, 2007), helping navigate gaming culture and establish oneself in it.

I'm not a skilled player, but I play using such difficulty levels that it's not a problem. On the other hand I know I've developed from when I started playing, and I'm happy for this development. Woman, 21

I feel like I'm a skilled player, because gaming has after all been my hobby for my whole life and I'm also competitive, so I've always sought to be better than others in games. If I for example start a new game, the game's genre doesn't matter, but I learn the game mechanics really quickly and get good at it. Man, 22

In PvE [player vs. environment] games you can learn so much and then you can be the smart one who knows everything and makes everyone else's experience much better because you're the dictionary for them. Man, 25

An interesting negative outcome of gaming skills for some respondents was that their high level ruled out potential people to game with. Skill- or rank-based multiplayer systems or large differences in skill between friends and spouses could make gaming together difficult or even impossible, limiting opportunities for shared gaming sessions. This is also an example of how the different dimensions overlap and influence each other, as in this case, the skill difference had an impact on the social aspects of playing.

I would like to play more often with my live-in partner, but the difference in skill levels sometimes makes it difficult. Man, 24

I have too high a rank to play competitive game modes with all of my friends. Man, 16

#### 4.2.6. Fantasy and immersion

Closely related to the dimension of escape and relaxation, the elements of fantasy and immersion common in gaming were an important part of gaming for some respondents. The strong emotions elicited by games (see Lankoski, 2012), the ability to figuratively cross the borders of everyday life, and the different immersive qualities of games (Ermi and Mäyrä, 2005) were all brought up in the responses.

I like gaming because I get to do things I couldn't do in the normie world. I like building houses and expressing myself in *The Sims* and *Minecraft*. I wouldn't necessarily get around to designing houses and interior decoration using a pen and paper at home, but in games it's easier. Woman, 20

Game fictions were an important part of the gaming experience, and while immersion can stem from different elements of game play (Ermi and Mäyrä, 2005), respondents mainly discussed imaginative immersion. Game fiction affords players opportunities to experience themselves and their everyday surroundings differently, and to toy with alternative ways and modes of being (Cremin, 2015). In addition to experiencing game stories in the games, some respondents engaged with them in other media (see Koskimaa et al., 2021) or expanded on the stories by writing their fanfiction, cosplaying as game characters, or drawing them (see *Creativity and expression*, below).

Several respondents compared their experiences with games to other media such as books and movies. Both similarities and differences were brought up in the responses. Echoing the notion of the *active audience* (see Behrenshausen, 2012), interactivity (see Christopher and Leuszler, 2023) was typically seen as the key difference.

I like vicarious experiences, adventure, surprises, and exploration, and it's not possible to get similar experiences from, e.g., books or movies. Although they can also be emotional, a good game brings your emotions to the surface in a more personal way. Woman, 24

These [story]games and moments in them are major memories for and will hopefully remain such even when I grow older. I think video games are the best form of art because they have one added layer of interactivity. Man, 16

#### 4.2.7. Creativity and expression

Although games are often discussed from the perspective of consumption rather than production, gaming also provided an important outlet for creativity and self-expression. For some respondents, creativity was expressed through game play itself, whether as designs in games such as *Minecraft* or *The Sims* or as innovative tactics in competitive games. Others enjoyed recording and sharing videos of their gaming.

I watch and make gaming streams. Sometimes gaming videos, but mostly I stream on *Twitch*. Man, 17

Activities such as cosplay, writing fanfiction, or drawing served as avenues for self-expression outside immediate game play contexts. They allowed participants to turn their interest and investment in games into a wider transmedia experience (see *Ways of engagement* below), expanding and re-interpreting characters and game stories, and, in the case of cosplay, rendering the digital into the tactile, allowing for new forms of engagement with the source material and constructing identity both as fans and as individuals (Lamerichs, 2011).

I also do cosplay like many of my close friends and we have cossed and planned to coss game characters from our favourite games. I've also drawn some fan art and written fanfic of game characters, but that's fallen by the wayside a little recently; now I mostly do it when I want to give my friends for example a card with a picture of their favourite character. Instead I've focused on getting both fanmade and official merch[andise] of games that are important to me, such as stickers, jewelry, hoodies, and comics. Woman, 25

A different form of self-expression, game creation and modification as a hobby is an established part of gaming (Sotamaa, 2010; Lai et al., 2021). Some respondents modified (“modded”) existing games, designed, and sometimes developed their own games as well. One respondent expressed his views on how gaming companies' control over their product was limiting players' sense of agency (see also Blom, 2022).

I've even spun out a couple of shitty games that are inside jokes with friends. Man, 17

Some developers also openly oppose modding which is also worrying. So the general trend is moving away from the player's decision making. Man, 22

#### 4.2.8. Game and genre attributes

Connecting to many of the dimensions discussed above, individual games and game genres were unsurprisingly an important part of the respondents' gaming (e.g., Bergstrom, 2019). While many respondents reported playing a wide range of games and genres, others focused on a single game or two. This dimension demonstrates how players do not necessarily enjoy all kinds of games or gaming in general—something that may be overlooked in contexts such as gamification or game-based learning (see Deterding, 2014). This said, many respondents enjoyed a diversity of different games and genres.

I play FPS games like *CS:GO, Apex Legends, Half-Life, Overwatch* and *Valorant* the most. After that I play simulation games like *The Sims* games, *Minecraft* and *Animal Crossing* the most. I tend to play a pretty diverse range of games, I also enjoy musical rhythm games, rally games and different story-driven games. Woman, 19

I'm not interested in single-player games and I usually get bored of those after a few hours of playing. Non-binary, 25

Games and genres could also deter players, as respondents also reported avoiding individual games or game types, typically competitive games. Often this was because of the hostility of their communities or perception of the prevalent mentality in them. Much like gaming is not just the act of playing a game, in this context, a game was not just the game itself but also the culture, meanings, experiences, and assumptions attached to it.

One category of games that I try to avoid is FPS games. I don't feel like I enjoy competition enough for games like *CS* [*Counter-Strike*] or *Overwatch*. Man, 22

I don't play online games almost at all (and if I do, I don't open the chat) because I know that in some people's view it's part of gaming culture to harass everyone assumed to be a women and minority co-players. I don't feel like these kinds of games could make me happy or help me relax. Non-binary, 24

### 4.3. Internal and external influences

According to the DGR theory (Sokka, 2021; Meriläinen, 2023), there are different types of external and internal influences that influence people's relationship to digital games, encompassing personal, social, institutional, and societal and cultural factors. Following this classification, we next discuss the influences through the subthemes of *External influences* and *Internal influences*, both consisting of several subthemes. By external influences, we mean everything that the respondents position outside of themselves (i.e., social, material, institutional, and societal and cultural factors), while by internal influence, we refer to those influences that respondents see as stemming from themselves (e.g., their personality, preferences, and needs). Like the dimensions of meaning discussed in the previous theme, these influences are not neatly separated but overlap and interact.

#### 4.3.1. External influences

External influences mentioned in the responses varied from those that increased or reduced the amount of engagement with video games to those that influenced the type of engagement respondents had with gaming. Similarly, they could be direct and forceful (i.e., parents forbidding certain games) or have a softer, more indirect influence (i.e., friends enjoying certain games).

##### Societal and cultural factors

Negative discourses about video games in public discussion both in Finland and globally, as well as the societal stigma around gaming that these discourses reinforce, were brought up by multiple respondents. Negative views on gaming did not typically influence the respondents' play activities, but rather how openly they would discuss their gaming. Earlier research has shown that people sometimes avoid talking about their gaming activities or identifying as a gamer due to gaming being perceived in a negative and stigmatized way (Shaw, 2012). Several respondents, however, also brought up that attitudes toward gaming had become more positive and accommodating.

Older relatives also often think of games as “children's stuff” and adults who play are perhaps considered a bit childish. I think it's important to be aware of the negative effects of excessive gaming, but often still in public discussion gaming is seen more often as a negative thing. So I would also like more public discussion about the positive side of games, e.g., about what you can learn from games and that games would also be appreciated as an art form. Woman, 25

Another prominent societal discourse that influenced engagement with gaming was the perceived hostility of game communities and gaming culture in general. Overlapping with the previously discussed *Game and genre attributes*, this could lead to avoiding certain games or game types altogether.

I don't avoid games, but I avoid game communities a lot. The communities of different games are often ungrateful towards the creators of the games and towards other players. Man, 17.

Respondents discussed avoiding certain game communities or regulating their engagement in these (for instance, by muting all communication channels) due to homophobic, transphobic, racist, and sexist comments and verbal abuse. With some of the women and non-binary respondents, this had a gendered dynamic. These respondents had experienced hostility due to their gender while playing and this had influenced the way they engaged with gaming (see Meriläinen and Ruotsalainen, 2022; Friman, 2022). Gaming cultures have long had issues with discrimination and hostility toward women and minorities (Kirkpatrick, 2017; Cote, 2018), yet our data also reveal a non-monolithic gaming culture: the majority of the respondents would not engage in or support hostile and sexist behavior, and many also actively resisted these behaviors and confronted those engaging in them (Meriläinen and Ruotsalainen, 2022; see also Nakamura, 2012).

I have lots of bad experiences of gaming. Most often these experiences are related to my gender. There's a lot of verbal violence towards women in the gaming world. I don't like playing team-based games with strangers because as soon as the co-players hear I'm a woman, calling me a whore and other misogyny starts. Woman, 21

##### Institutional factors

The institutional factors affecting respondents' activities around video games varied from family to school, work, and hobbies. In the case of the family, the main influence was the parents. This would mainly manifest in the ways parents would set rules and limitations on the time spent playing, and the types of games played (see Kutner et al., 2008; Meriläinen, 2021).

Well, I wasn't even allowed to play *Minecraft* until I was 12 years old, because my parents thought it was too violent as you can kill cows and pigs in it. Nowadays, when I no longer live with my parents, there are of course no restrictions. They have sometimes wondered that I play, say, *GTA V*, when they don't think it sounds like my kind of game at all. Woman, 20.

Hobbies, school, and work were mainly mentioned by respondents due to the time constraints they would introduce to playing video games. Related to this, some respondents mentioned that they would like to have some more time for playing video games or that they used to have more time to play video games. The changing rhythms of everyday life and engagements influenced how much the respondents spent time engaging with video games (see Apperley, 2010; Meriläinen, 2022).

Nowadays the only thing limiting my gaming is myself. The time I spend on gaming has decreased because of studies and a relationship, but I still prioritize time for my gaming, especially on weekends. Sometimes I'd like more time off from my busy life for gaming. Man, 25

##### Social factors

Friends and social circles are an important part of young people's life, influencing the way they spend their time, form their identities, and construct their values (Youniss and Haynie, 1992; Oransky and Marecek, 2009). Friends also had a considerable influence on our respondents' gaming activities. Some respondents expressed a lack of friends playing some of the games they would like to play or a general lack of gaming friends.

I'd like to find more people to game with. In one community there's always at least people to talk with, but people partially play other games than I do, so I don't get to play enough games that interest me with people I know. It would also be great to find women or others [likely referring to gender] to game with. Woman, 22

Other respondents mentioned how they would play certain games only with friends or because friends would want to play them, thus possibly even changing their usual gaming preferences to maintain their social circle. Multiple respondents would divide their preferred games into two different categories: games they played alone and the games they played with friends. As per Eklund (2015), who we play games with affects not only what is played but also how the games are played.

I play alone and with my boyfriend. I play *Witcher 3* alone and *Redecor* home decoration game on my mobile phone. I play single-player games with my boyfriend and sometimes split screen co-op games. Woman, 25

##### Material and resource factors

Material and immaterial resources also influenced the way young people engaged with video games. Particularly common were financial limitations, manifesting in the state of gaming equipment: sometimes the respondents could not afford to buy new games or upgrade their gaming computer. Financial constraints also manifested as some respondents only bought games that were on sale or playing free games. While the questionnaire did not address the respondents' socioeconomic status, responses showed obvious differences in personal finances.

As I'm an adult and live alone I have money for games and devices just fine. As a kid I couldn't get too many of them in a year, if I had to save from my fiver [5 euro] weekly allowance. I didn't buy even many cheap games because it was so expensive if they cost say 10€. At least I learned the value of money. Man, 25

I don't have enough money for games and I'm constantly wondering if I can buy an interesting game or if I have to limit my food purchases. The games are constantly getting more expensive, even though it seems that, for example, the people who actually made the game in the game development team do not get enough compensation for the profits of all the games for the investors and those who have not made the game themselves. This seems unfair. I also don't think I have time to play enough because of my studies and running my everyday life. Non-binary, 21

This highlights how gaming can also be a constant negotiation of how one can participate with limited resources, whether these relate to material (the gaming hardware), personal finances, or available time (e.g., Apperley, 2010; Meriläinen, 2022). Gaming demands some expenditure of time and money by necessity, meaning that it competes for those limited resources with other activities.

#### 4.3.2. Internal influences

In addition to external influences, respondents also mentioned internal influences that affected their engagement with video games. Alongside the personal meanings discussed previously, we identified two distinct subthemes, one related to game accessibility and the other to worries over excessive gaming.

##### Accessibility

The previously discussed skill requirements of game play can present an issue of accessibility, whether because of lacking experience with a particular kind of game, intentional game design, or disability (e.g., Baltzar et al., 2022). Players' perception of their skill could encourage or discourage them from playing games or influence game choices, while some respondents had physical limitations to their gaming.

The obstacle with some games is their difficulty, which is often related to a lack of patience to grind the characters forward. I also feel that I own more games and devices than I have time to play. Often it is chosen by a familiar and safe game that you don't have to think too much about, instead of grabbing a more challenging game that is waiting for its turn to be advanced in. Woman, 23

Console gaming is practically impossible due to chronic tendinitis. All controllers are from the same mold. Woman, 25

##### Worry over excessive gaming

Rather than gaming being limited by the constraints of other hobbies, work, and responsibilities, some respondents would actively self-regulate their gaming for wellbeing reasons. Some considered their own gaming habits excessive now or in the past and consciously wanted to game less. This was sometimes a source of distress and worry over one's mental health and the risk of addiction.^2^

Although the subject of problematic gaming was not very prominent in the data, mentions of occasional excessive gaming or unhealthy gaming habits featured in many responses. Problems and concerns related to excessive gaming are an everyday feature of gaming and something players are aware of, but our data also suggest that from the perspective of the topic's relevance to young people, research on problematic gaming appears dramatically overrepresented in the current literature.

When I was younger I may have played too much, so that, e.g., I did less of other things in my free time and I was pretty addicted at times. In time it got better and with age my approach to gaming became more casual. Now I play a lot and enjoy it, but I understand that real life is more important. Man, 19

[Gaming] is not important but I've been a bit of an addict for it for over a decade … Back in the day gaming sure was a problem as I couldn't concentrate properly on other things, e.g., looking for work or even eating. Man, 24

### 4.4. Ways of engaging with gaming

Young people continuously form relationships with video games that go beyond playing games. This is unsurprising, as video games today are often large transmedial products; either there is an element of transmedial storytelling to the product, the story is told through multiple mediums (Jenkins, 2006), or there is a shared world across multiple mediums (Tosca and Klastrup, 2019). Often this means that a product or franchise consists of multiple different artifacts, such as games, films, comics, and collectibles, rather than just a video game. Designing video games in a transmedial way has long been a part of gaming culture (see Koskimaa et al., 2021), and these design choices encourage multiple modes of participation.

These different modes of participation with video games, their transmedial content, and ultimately with gaming culture at large can be understood through four interconnected fields described in the DGR theory: *Personal gaming, Following gaming cultures, Production of gaming cultures*, and *Consumption of meanings of gaming cultures* (Meriläinen, 2023).

#### 4.4.1. Personal gaming

Personal gaming can be seen as the activity of playing alone and solely engaging with the game rather than other mediums and meanings conveyed through them. Indeed, some of our respondents would explicitly mention that their gaming relationship consisted exclusively or almost exclusively of playing video games.

I often discuss games with friends but otherwise the gaming hobby is largely limited to just playing. Men, 20

Gaming is its own thing in my life and other things are their own. Man, 22.

#### 4.4.2. Production of gaming cultures

Production of gaming cultures functions at multiple levels: institutional, social, and personal. Those creating video games are an obvious example of the production of gaming cultures, but as our data show, players also continuously participate in this production. Gaming culture production encompassed a wide variety of different activities and forms of engagement from writing game reviews to cosplaying game characters. These modes of engagement would allow challenging the normative production of gaming cultures, shifting the focus away from what is traditionally held important in Western gaming cultures (namely, skill and mechanics, see Kirkpatrick, 2017; Ruberg, 2018), to alternative themes. For instance, one of our respondents discussed her relationship with games and cosplaying by highlighting the importance of fantasy worlds and the possibility of momentarily existing within these worlds (see Lamerichs, 2011).

What's important to me in games is their world, the opportunity to be someone else, and the sense of community. The fantasy worlds that are important to me in games are something to immerse yourself in for a while, sometimes to escape from reality and sometimes to experience adventures that you could never encounter in the real world. You can be someone else for a while, without changing yourself in any way. Same with cosplay; for a while we are part of another world, a story, a community, an adventure. Woman, 25

Different levels of production of gaming cultures are not separate categories, but a continuum that can be examined on the scales of professionalization and labor. Video game content creation can be seen as a form of labor, often discussed through the term *playbour* (Törhönen, 2021). Content creators are often not paid for this labor and it is quite common that they do not expect to be compensated either. However, there was also present an interesting continuum of ways of engaging and producing content for games or even full games for free as a hobby and simultaneously having a job related to games.

I watch streams and game videos, make my own games in my free time and partly work with game education. I am also involved in the activities of the local board game association. Man, 25

#### 4.4.3. Following gaming cultures

According to Koski (2008), the most common form of following sports is spectator sports. The closest analogue to this in gaming cultures are esports which are becoming both increasingly popular and more institutionalized in the vein of traditional sports (Brock, 2017; Scholz, 2019; Törhönen, 2021; Ruotsalainen, 2022). One respondent described both following esports and having esports streams on as “background noise” rather than something fully engaged with.

I have always followed sports extensively, and competitive games have also come into this palette over time. Nowadays, this especially includes *CS* [*Counter-Strike*, referencing streams], which is often just a background noise, and sometimes I watch games much more closely … I no longer play the games I follow the most (*CS, LoL*), so my following is specifically focused on the competitive side, but in the past, when I played those games, I also followed some side content. I occasionally listen to podcasts related to competitive games. Man, 25

Echoing earlier research on engaging with games beyond playing them (Koskimaa et al., 2021), watching videos and live streams were the most popular ways of following transmedial content about games. These different modalities (listening and watching) allowed respondents to engage with games differently than just playing games themselves, also changing where and how the game-related content could be followed. Sometimes the act of playing games was a side activity, for example, watching television. This problematizes the construction of players in general as an “active audience,” in comparison to more “passive audiences” (see Behrenshausen, 2012), and highlights how there can be different intensities of engagement depending on context and activity.

Following gaming cultures sometimes also happened in relation to changes in playing routines, as previously discussed under the main theme of *Level of engagement*. With age, consuming content could become more common than playing video games in respondents' lives, presumably at least in part because of convenience.

#### 4.4.4. Consumption of meanings of gaming cultures

Consumption of meanings of gaming cultures can happen through consumption of both material (games, gaming devices, and game merchandise) and immaterial (fanart, stories, and attitudes) things. What is central is that it broadens the meanings beyond individual artifacts and frame activities through participation in gaming culture. Echoing different levels of participation in gaming, consumption of meaning often has a level of intensity or engagement that following gaming culture does not necessarily have. One way to conceptualize the difference between consumption and following is comparing a sports fan and a sports spectator: a sports spectator is someone who merely watches the games, while a fan is invested in the game and usually its teams and players (Wann and James, 2018).

My friends and I share experiences about the games we play. I have also bought books or small decorative items related to my favourite games. Woman, 23

Consuming meanings is also often a social activity and socializing was part of most of the gaming activities discussed by our respondents. Even when one is not directly socializing, they can be contributing to the co-construction and upkeep of the social world of gaming by, for instance, streaming game play, participating in a multiplayer game, or posting about games on social media.

When examining engaging with games and socializing from a transmedia perspective, certain mediums were mentioned for social interactions by our respondents. *Discord* was mentioned for communicating during playing video games, but also for maintaining and hosting communities around gaming. *Reddit* was mentioned as a place to both participate in and follow discussions about games on social media. *Twitch* was used for multiple purposes by our respondents: to create content by streaming, to consume content by watching streams, and also to host communities around particular streamers. Not all social interactions took place online, as LAN parties, gaming bars, and hobby associations also allowed respondents to socialize.

## 5. Discussion

In our discussion, we first highlight different continuums or spectra that can be used to explore youth gaming and address its diversity. We then discuss agency and the positioning of youth in relation to gaming in research.

### 5.1. Understanding young people's gaming

Although gaming can certainly have both beneficial and detrimental impacts on game players, these are not the be-all and end-all of young people's gaming culture participation, nor did the young people in our study usually frame gaming primarily as a balancing act between the two. The spectrum of beneficial–harmful is only a single, narrow perspective on young people's participation in the social world of gaming. Providing alternatives to the impact point of view is crucial to how we view games and gaming as culture, as media, and as a part of everyday life. The framing of gaming influences perceptions and attitudes (Kümpel and Haas, 2016), and whether gaming is primarily seen, for example, as a tool, a health risk, a form of art, or an everyday activity impacts how it is perceived, discussed, and studied. Furthermore, knowledge of the different potential impacts of gaming without a broader understanding and critical exploration of the phenomenon can cause its own problems, whether in the form of moral panics (Pasanen, 2017) or unrealistic optimism (Deterding, 2014).

As shown by our analysis, what is often referred to with the shorthand “youth gaming” is a hugely diverse and multi-faceted phenomenon. For each individual player, a multitude of factors come together to produce a personal, complex, and sometimes conflicted relationship with the social world of digital gaming. Based on our results and informed by the DGR theory approach, we suggest ten other intersecting and interacting continuums or dimensions that can be used instead or alongside explorations of beneficial vs. detrimental when making sense of young people's gaming. Based on our results, we present the following key framings for conceptualizing and understanding youth gaming and its complex dynamics. Our listing is not an exhaustive list, nor a foundation for a categorical model (see Meriläinen, 2023). Instead, it is intended to summarize our results and suggest positioning and perspectives for future research.

#### 5.1.1. Production–Consumption

While engaging with gaming culture, young people are constantly taking part in both its consumption and its production. These can take very concrete forms, as demonstrated by our respondents organizing events, making and watching gaming videos and streams, and purchasing games and merchandise. Production and consumption can also take more abstract forms, as youth participate in the co-construction of the social world of gaming in countless everyday game play exchanges, discussions, and social media posts, in a process of simultaneous production and consumption.

#### 5.1.2. Leisure–Labor

The professionalization of different gaming activities such as streaming or competitive gaming blurs the lines between work and play (e.g., Brock, 2017; Törhönen, 2021), prompting and forcing youth to navigate and find their place in an intensely commercialized ecosystem and make decisions on potentially turning a hobby into work. However, the distinction between leisure and labor also relates to gaming more generally. Learning how to play games often requires considerable effort, and game play can in itself be laborious and repetitive (e.g., Orme, 2021). This framing also connects to fundamental societal discussions on the value of play and leisure, seen, for example, in some of our respondents' use of professionalization to justify gaming.

#### 5.1.3. Stranger–Insider

The continuum from stranger to insider (see Unruh, 1979; Meriläinen, 2023) provides important insight into engagement with gaming cultures. While self-definitions such as “casual” and “hardcore” appeared in some individual responses, they capture only a part of participation (see Vahlo and Karhulahti, 2022). The continuum connects to existing discourses (e.g., Shaw, 2012; Paaßen et al., 2017) of gamer identity and gaming culture insiderness as well as fluctuations in an individual's relationship with gaming (Jiang, 2018; Bergstrom, 2019; Wiik, 2023). As the social world of gaming is massive, it is inevitable that an individual is a different kind of participant in regard to different aspects of gaming, and that this participation changes with time: no one starts as a regular or an insider.

#### 5.1.4. Private–Public

The continuum from private to public relates to different aspects of youth gaming: the situating of gaming devices and parental mediation, game and gaming preferences, streaming, community participation, self-expression, and the role that gaming occupies in an individual's social life, to name a few. As shown in our results, keeping gaming private can stem from a personal preference for cozy solitary relaxation, but it can also be the result of a fear of ridicule or discrimination, or the lack of friends. Gaming also allows players to exist somewhere between private and public, such as when publicly participating in games and communities anonymously, allowing, for example, experimentation with different social roles and identities. Different popular platforms which facilitate gaming and activities around gaming, such as *Twitch* and *Discord*, also often create locations that are neither fully public nor fully private.

#### 5.1.5. Allowed–Forbidden

Closely related to the continuum of private–public, there are important power dynamics that influence youth gaming. Direct parental control (e.g., Meriläinen, 2021), negative societal views, and stereotypes that relate to gaming (e.g., Kowert et al., 2014; Latinsky and Ueno, 2021), as well as struggles over who is allowed to exist or belong in game cultures, exemplified by different discriminatory behaviors (e.g., Paaßen et al., 2017; Ortiz, 2019; Friman, 2022), exert their influence on young game players by affording or removing gaming opportunities. As discussed in some of the responses, factors such as disability can also push individuals away from games if not accounted for when games and devices are designed (e.g., Baltzar et al., 2022).

#### 5.1.6. Inclusion–Exclusion

In addition to different intersectional variables such as race, gender, disability, and class, there are different phenomena such as the meritocratic ideals especially prevalent in competitive gaming (Siutila and Havaste, 2019) or normative hostility (Hilvert-Bruce and Neill, 2020) that include some young people in gaming and exclude others. These structures and phenomena are present in everyday interactions; as key game culture participants, young people both construct and dismantle barriers to participation and are included and excluded by them. Problems of inclusion and exclusion can also come about through skill differences, as players' different levels of skill can make it difficult or even impossible to play certain games together. Exclusion can also stem from lacking resources (i.e., not affording to buy games and gaming devices), tying into larger societal issues of inequality (Apperley and Gray, 2020).

#### 5.1.7. Casual–Intense

Games can be played with different mentalities and social goals (Juul, 2010; Kallio et al., 2011), and our respondents had diverse approaches to how they played games. Casual in this context does not refer to game types but to mindsets: a player can play a very simple game very intensely (see Deterding, 2019) or a complex game very casually. The mentalities do not necessarily reflect an individual's overall relationship with gaming, but instead fluctuating preferences and different contexts. The difference between casual and intense does not by default equate to a difference between having fun and gaming seriously—although some of our respondents also discussed this—as both our study and previous research (e.g., Ryan et al., 2006; Vahlo, 2018) show that for many players, the experience of enjoyment stems from achievement and competition. The level of intensity does not only relate to gaming, as it is possible to participate very intensely in some aspects of gaming while taking a very casual approach to others (e.g., Orme, 2021).

#### 5.1.8. Mundane–Special

Gaming is inevitably interwoven with other aspects of a player's life, and has to be negotiated in relation to mundane everyday commitments such as studies, sleep, social relations, and work, and takes place regulated by the constraints of resources such as time and money (e.g., Pargman and Jakobsson, 2008; Apperley, 2010; Meriläinen, 2022). As discussed by the youth in our study, gaming can be boring, but it can also provide exceptional experiences, whether through escapism and immersion in game stories or memorable moments with friends or family. Whether gaming is viewed as a mundane and domesticated part of everyday life or as distinct from, it also influences how it is approached in public discourse or contexts such as parenting.

#### 5.1.9. Focused–Broad

Acknowledging diverse forms of gaming culture participation, such as cosplay (Lamerichs, 2011) or watching games instead of playing (Orme, 2021), recognizes that one can form a relationship with games in a multitude of ways and contests the narrow conception of “gamer” and defining gaming culture participation around it (see Consalvo, 2007). Perceiving gaming culture participation only or primarily as playing games privileges certain types of activities, and consequently can end up privileging those who have historically had access, both in terms of practice and identity, to video games and gaming cultures, typically white middle-class men (Kocurek, 2012; Shaw, 2012; Fletcher, 2020).

#### 5.1.10. Local–Global

Although in this article we have discussed *gaming culture* for the sake of convenience, essentially gaming takes place in culture rather than being a separate entity (Shaw, 2010). It follows that young people's gaming is inevitably shaped by the same factors that shape their lives overall: societal structures, demands, and attitudes, cultural affordances, parents' and peers' views and attitudes, different intersectional positions, and as demonstrated by the COVID-19 pandemic (e.g., Bengtsson et al., 2021; Cote et al., 2023), global phenomena. Alongside the macro scale societal and cultural factors, it is equally important to pay attention to the micro-scale influence of individual and local factors. The generic young person exists only as an abstraction in research.

When we combine the diversity of young people, the diversity of games as transmedia, the diversity of gaming culture participation in terms of both intensity and ways of engagement, and the diversity of individual instances of gaming, the complexity of youth gaming becomes apparent. We must critically consider how accurate and truthful image results from reducing the phenomenon to a selection of variables. Qualitative approaches have repeatedly (e.g., Nielsen, 2016; Russell and Johnson, 2017; Bengtsson et al., 2021; Meriläinen, 2021; Zhao and Zhu, 2021) brought up underexplored facets of youth gaming crucial to understanding it, yet the field continues to be dominated by risk-focused quantitative studies. Research perspectives focused on the outcomes of gaming detached from its wider context provide limited help for understanding young people's gaming, or gaming in general. Philosophical questions of value also come into play: is a memorable experience from a game or a fun moment with friends valuable as such, or because it contributes to something else, such as wellbeing? The two are not mutually exclusive, but the focus has firmly been on the latter.

### 5.2. Young people are subjects, not objects

Attitudes toward gaming and gaming cultures appear to be slowly shifting, yet seeing games in a very polarized way has a long history that still influences contemporary discourses about gaming (Rogers, 2013). Shaw (2010) notes that in media, gaming has been largely depicted as an undesirable activity, and likewise those playing video games (see Kowert et al., 2014). This goes hand in hand with how videogames have long been targeted by moral panics and viewed as a source of aggression, moral decay, and addiction (Rogers, 2013; Pasanen, 2017).

Questions of young people's agency in relation to different media have long been a core topic in discussions of media literacy (e.g., Buckingham, 1998; Hobbs, 2011) and are central to the contemporary debate on the role of digital media in young people's lives (e.g., Granic et al., 2020; Vuorre et al., 2021). Acknowledging this agency is important not just in terms of understanding the phenomena but also to bridge disconnects among theory, measurement tools, and young people's experiences (see Nielsen, 2016): gaming does not happen to young people, but is something they choose to do. The approach taken in this article positions young people as active agents who engage with and participate in gaming, use (and sometimes abuse) games, and influence, create, and critically examine gaming cultures.

Obviously, we do not advocate against studying the impacts or outcomes of gaming, as the importance of detailed knowledge on them is apparent: it has been well-documented that games can cause and tie into health and wellbeing problems (e.g., Snodgrass et al., 2014; Männikkö et al., 2017; Shi et al., 2019; Hamre et al., 2022) and, for example, promote learning and cognitive development (e.g., Moisala et al., 2017; Martinez et al., 2022) and many of our respondents also discussed both negative and positive outcomes. However, whether intentionally or not, a focus solely on impact can end up ignoring much of what makes gaming meaningful and important for the young people themselves, and render gaming primarily a utilitarian issue, its value typically defined from the outside and its relevance derived from its measurable outcomes. This can end up erasing young people's agency: instead of exploring how young people *do* gaming and express, explore, and become themselves through and in gaming cultures, the focus turns to what gaming does to young people, rendering subjects into objects and active agents into victims or beneficiaries. Examining outcomes and meanings are luckily not mutually exclusive. On the contrary, combining different approaches in terms of both research philosophy and research methods likely yields more holistic answers to complex questions of positive and negative outcomes.

Outside of academia, many parents and professionals encounter, celebrate, and worry over young people who enjoy gaming. In these different situations and contexts, an understanding of how gaming is intertwined with other aspects of young people's lives, from self-expression to time spent to structural oppression, can be immensely valuable. It can mean the difference between parents being emotionally supportive or dismissive (see Bax, 2016; Meriläinen, 2021) or between a professional focusing on causes or on symptoms (see Nielsen, 2016). The DGR theory can also help individuals, regardless of their gaming participation, critically examine their own relationship with digital gaming and the different factors that shape it (see e.g., Przybylski, 2014; Russell and Johnson, 2017; Hopia et al., 2018).

### 5.3. Limitations and strengths

Despite the rich data, it was apparent that some respondents had only answered the assisting example questions rather than going beyond them, limiting their responses. The assisting questions were added conscious of the possibility of this happening, as it was considered preferable to getting very short answers or the respondents misinterpreting the broad questions (see Braun et al., 2021). The cultural diversity of the respondents was limited, with ethnic and cultural minorities only marginally present. Because of our self-selected sample, our study is not representative of the diversity of young people who participate in gaming, although in our estimation, it likely reflects the experiences of typical, regular game players in Finland quite well. This said, people at opposing ends of the gaming spectrum, very intense, professional, or problematically gaming players as well as uninvested, occasional players are present in the data only to a limited extent.

As a qualitative study of young people's gaming through a reasonably large sample, our study is to our knowledge the first of its kind in Finland and provides important new insights into the subject. The responses suggest that many young people expressed themselves quite freely—something that might not have been possible in the social context of a face-to-face interview. By drawing on young people's own experiences instead of using standardized quantitative measures, we have highlighted aspects of gaming that are essentially not measurable but are nevertheless integral to understanding it.

## 6. Conclusion

Using the DGR theory, we illuminated the diversity of young people's gaming as a phenomenon influenced by a great number of variables, complicating the dichotomy of gaming being either beneficial or harmful. Based on our results, we suggested alternative and complementary perspectives for future explorations of youth gaming. Finally, we drew attention to the importance of acknowledging youth as individual active agents, capable of complex reflection on gaming culture phenomena when discussing and studying young people's gaming.

## Data availability statement

The datasets presented in this article are not readily available because of privacy considerations, but will be made available through a public repository later for further research use once anonymized. Requests to access the datasets should be directed to MM, mikko.merilainen@tuni.fi.

## Ethics statement

Ethical review and approval was not required for the study on human participants in accordance with the local legislation and institutional requirements. Written informed consent from the participants' legal guardian/next of kin was not required to participate in this study in accordance with the national legislation and the institutional requirements.

## Author contributions

MM collected the data used in this study. MM and MR contributed equally to the analysis and writing. All authors contributed to the article and approved the submitted version.

## Footnotes

^1^The word for free play in Finnish is *leikki, paidia* as opposed to *ludus* following Caillois (2001/1958; cf. Masek and Stenros, 2021) formulation.

^2^We use the word “addiction” here as it was the word typically used by the respondents (cf. Nielsen, 2018).

## Supplementary material

Supplementary File 1 Questionnaire.
