# Supplementary material for: The light, the dark, and everything else: making sense of young people's digital gaming
Source: Front Psychol. 2023 Jun 12;14:1164992. doi: 10.3389/fpsyg.2023.1164992 (PMC10306168; doi:10.3389/fpsyg.2023.1164992)
Supplement: Supplementary File 1 — Questionnaire. [file Data_Sheet_1.pdf]

Appendix 1. Qualitative questionnaire. Translated from Finnish by the authors.

Background questions

Age (open field)

Gender (woman / man / non-binary / don't know or do not want to disclose)

Area of residence (Capital area / Southern Finland (not including capital area) / Central Finland / Western Finland / Eastern Finland / Northern Finland)

[voluntary question] **Which of the following describe you (you can choose multiple)**

- I was born in Finland
- I was born outside Finland
- One or both of my parents were born outside Finland
- I belong to a language or culture minority, which? (open field)

Main questions (additional questions in parentheses)

Tell us about your gaming (e.g.: What games do you play? Who do you game with? Why do you play games? Where do you play games? When or in what situations do you play games? Do you take part in competitive gaming? What devices do you game with? Is gaming important to you? Have you learned something by gaming? Where and how do you acquire your games?)

What else is gaming to you apart from playing games? (e.g. Do you watch or make streams or videos? Do you read gaming magazines? Do you mod or create games? Do you talk to your friends about gaming? Do you cosplay, draw game characters or write fanfic? Do you participate in social media gaming communities? Do you work or study with games or dream of doing so? Do you go to gaming events or organize them?)

How do you behave in games and gaming communities? (e.g.: Do you pay attention to your own behaviour? Do you encourage or help other players? Do you troll, grief or trash talk other players for fun? Do you play differently with people you know and strangers? Do you talk to other players? Do you lose your nerve easily? Do you address inappropriate behaviour? Do you cheat in games? What do you do if there are conflicts in-game?)

What aspects of gaming are the best, the most valuable or important to you personally? (e.g.: Have you made new friends? Have games offered special experiences? Are there important memories related to gaming? Do you think you are a skillful player? Do you have favourite YouTubers, gamers or streamers?)

Does something limit your gaming? (e.g.: Do you have enough people to play with? Do your parents set limits on your gaming? If your gaming is limited, do you circumvent these limits? Do you avoid some games or gaming communities? Do you think you have enough money for games and gaming devices? Do you have enough time for gaming?

Do you have bad experiences of gaming, or does something about gaming annoy or worry you? (e.g.: Have you encountered harassment or inappropriate behaviour? Do you think game culture has developed in a better or worse direction in recent years? Have you bullied other players? Has gaming

caused arguments or other problems in your everyday life? Do you think you play or have played too much?)

How do you think gaming is viewed and what do you think of these views? (e.g.: How do your parents view your gaming? Have you encountered gaming in school or youth work? Have educational or healthcare professionals commented on your gaming? How do you feel gaming is viewed in society more broadly? Is there something related to gaming that you think is talked about too much or too little, for example in the media, on social media or in education?)
